# All-optical field-free three-dimensional orientation of asymmetric-top molecules

**DOI:** 10.1038/s41467-018-07567-2

**Published:** 2018-12-03

**Authors:** Kang Lin, Ilia Tutunnikov, Junjie Qiang, Junyang Ma, Qiying Song, Qinying Ji, Wenbin Zhang, Hanxiao Li, Fenghao Sun, Xiaochun Gong, Hui Li, Peifen Lu, Heping Zeng, Yehiam Prior, Ilya Sh. Averbukh, Jian Wu

**Affiliations:** 1 State Key Laboratory of Precision Spectroscopy, East China Normal University, 200062 Shanghai, China; 2 AMOS and Department of Chemical and Biological Physics, Weizmann Institute of Science, 7610001 Rehovot, Israel; 3 Collaborative Innovation Center of Extreme Optics, Shanxi University, 030006 Taiyuan, Shanxi China

## Abstract

Orientation and alignment of molecules by ultrashort laser pulses is crucial for a variety of applications and has long been of interest in physics and chemistry, with the special emphasis on stereodynamics in chemical reactions and molecular orbitals imaging. As compared to the laser-induced molecular alignment, which has been extensively studied and demonstrated, achieving molecular orientation is a much more challenging task, especially in the case of asymmetric-top molecules. Here, we report the experimental demonstration of all-optical field-free three-dimensional orientation of asymmetric-top molecules by means of phase-locked cross-polarized two-color laser pulse. This approach is based on nonlinear optical mixing process caused by the off-diagonal elements of the molecular hyperpolarizability tensor. It is demonstrated on SO_2_ molecules and is applicable to a variety of complex nonlinear molecules.

## Abstract (web summary)

Alignment and orientation of the molecules allows studying the photon-molecule interactions in greater detail. Here the authors demonstrate the three-dimensional orientation of SO_2_ molecules in using COLTRIMS and orthogonally polarized laser pulses but in the absence of DC field.

## Introduction

Over the years, several optical approaches have been used to define preferred directions in space along which the molecules can be aligned or oriented (for a review, see refs. ^1–4^). Many applications rely on such manipulations providing detail for the study of photon−molecule interactions including high harmonics generation^5,6^, molecular orbitals tomography^6–8^, control of molecular photoionization and dissociation processes^9–11^, production of molecular movies with the help of X-ray free-electron laser sources and ultrafast diffraction of relativistic electrons^12–14^. Early on, intense nonresonant laser fields were combined with weak electrostatic fields^15–19^ to induce symmetry breaking along one of the laboratory axes. This was followed by introduction of single-cycle THz pulses^20–25^, alone or in combination with optical pulses^26–29^. Nonresonant phase-locked two-color laser pulses were used for inducing orientation of linear molecules via the nonlinear interaction with diagonal elements of molecular hyperpolarizability^21,30–36^.

Previous works were mostly focused on orientation of simple, linear molecules. Nowadays, efforts are being made to induce orientation of more complex molecules. Complete control over the absolute spatial orientation of the molecules may allow direct imaging of structure of gas-phase molecules^37^ using advanced free-electron laser beams with extremely high spatio-temporal resolution. A combination of DC field and long sharply truncated optical pulse was proposed as an efficient tool for orientation of asymmetric-top molecule under laser-field-free conditions^38,39^ and more recently, pulsed laser fields with twisted polarization were shown to be effective for partial enantio-selective orientation of chiral molecules^40–42^.

Here, we report the experimental demonstration of all-optical field-free orientation of asymmetric-top molecules using phase-locked Orthogonal Two-Color (OTC) laser fields, consisting of fundamental wave (FW) and its temporally overlapping second harmonic (SH). In our experiments, FW aligns the major molecular axis (the one with the highest polarizability) along the polarization direction, while the two fields together couple to the molecule via the off-diagonal components of the molecular hyperpolarizability tensor. This interaction leads to breaking of the azimuthal symmetry and to orientation of the minor molecular axis (the one with the second highest polarizability) after the OTC pulse is over, resulting in the field-free molecular orientation. The same principle applies to other nonlinear molecules similar to SO_2_, but with different arrangements of the polarizability axes. We explain the orientation mechanism in detail and explain how to choose the relative intensities (FW vs. SH) in order to orient a given type of molecule.

We introduce and experimentally verify this OTC orientation scheme. In Supplementary Note 2, we discuss a generalization of this scheme, and show how a combination of the OTC pulse with an additional linearly polarized laser pulse induces orientation in an ensemble of molecules lacking any symmetry, for example the propylene oxide (the first chiral molecules detected in interstellar space^43^).

## Results

### Experimental setup

We begin from the description of experimental setup, then provide a conceptually simplified one-dimensional model that illustrates the principles of our method, discuss the results of a more sophisticated fully three-dimensional simulation of the experimental measurements, and finally show the results of our experimental observations.

**Fig. 1:**
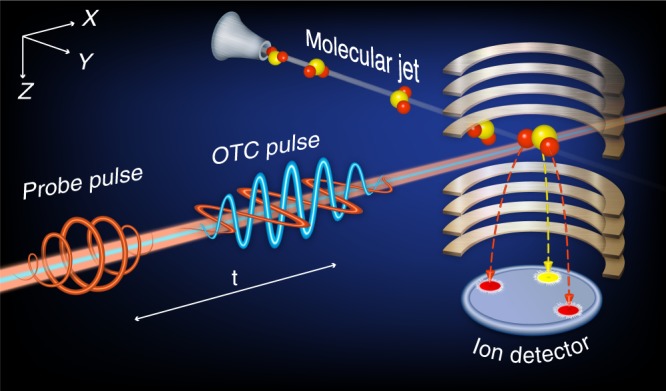
Layout of the experiment. A supersonic gas jet of SO_2_ molecules subject to a pair of synchronized two-color laser pulses with orthogonal polarizations in an ultrahigh vacuum chamber of COLTRIMS (COLd Target Recoil Ion Momentum Spectroscopy)

**Fig. 2:**
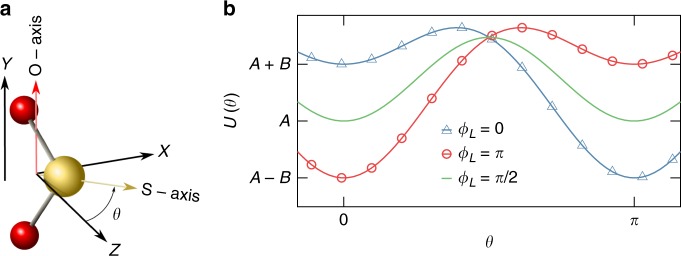
One-dimensional model geometry. **a** SO_2_ molecule, the atoms are color coded: sulfur—yellow, oxygen—red. The major axis is perfectly aligned along the laboratory *Y* axis. The minor axis lies in the *XZ* plane at an angle *θ* relative to the *Z* axis. **b** Potential energy as a function of *θ* for *ϕ*_*L*_ = 0, *π*, *π*/2 (see Eqs. 3 and 4)

In our scheme, the laser pulses propagate along the *X* axis. The FW is a *Y*-polarized femtosecond laser pulse with wavelength of 790 nm and the second color field is a *Z*-polarized SH pulse at 395 nm. The SH field temporally and spatially overlaps the FW, and is phase locked to it. The OTC pulse is focused on a supersonic molecular beam propagating along the *Y* axis. At a variable delay after the application of the two-color pulse, an intense circularly polarized probe pulse is employed to explode the molecules and to image their 3D spatial orientation via coincident Coulomb explosion imaging technique, as is schematically shown in Fig. 1. The experiments were performed on sulfur dioxide molecules (SO_2_) at rotational temperature 18 K. The *O*-axis is the major molecular axis with the largest polarizability, the *S*-axis bisects the bond angle between the oxygen atoms, and it is the minor axis with the second highest polarizability (see Fig. 2a). The molecular permanent dipole moment is directed against the *S*-axis. Driven by the ultrashort laser pulses, the reaction microscope of Cold Target Recoil Ion Momentum Spectroscopy (COLTRIMS) setup^44^ provides direct access to the spatio-temporal molecular dynamics with femtosecond time-resolution. The details of the experimental system are given in the Methods.

### Classical model

To illustrate the two-color orientation mechanism, we start with a classical ensemble of cold SO_2_ molecules whose major axis is perfectly aligned along the laboratory *Y* axis with a uniform angular distribution of the molecular minor axis in the *XZ* plane. The angle of rotation around the alignment axis is denoted by *θ*; see Fig. 2a. At time *t* = 0, a short phase-locked OTC pulse is applied to the ensemble. The electric field in the laboratory frame is given by

1 $${\cal E} = {\cal E}_1(t){\mathrm{cos}}(\omega t){\mathbf{e}}_Y + {\cal E}_2(t){\mathrm{cos}}(2\omega t + \phi _L){\mathbf{e}}_Z,$$

where **e**_*Y*,*Z*_ are the unit-vectors along the corresponding laboratory axes, ${\cal E}_i(t)$ are fields’ envelopes, *ω* is the carrier frequency of the FW field and *ϕ*_*L*_ is the relative phase between the FW and SH fields. The potential, *U* that describes the interaction of the laser field with the molecules (to third order in the electric field), is given by^45,46^

2 $$U = - \frac{1}{2}{\bf{\alpha }}_{ij}{\mathbf{E}}_i{\mathbf{E}}_j - \frac{1}{6}{\bf{\beta }}_{ijk}{\mathbf{E}}_i{\mathbf{E}}_j{\mathbf{E}}_k,$$

where **α**_*ij*_ is the polarizability tensor, **β**_*ijk*_ is the hyperpolarizability tensor, and **E** is the electric field. The tensors **α**_*ij*_ and **β**_*ijk*_ are symmetric in all indices^45^ and summation over the repeated indices is implied. For molecules having *C*_2v_ symmetry the hyperpolarizability tensor has three independent elements *β*_113_, *β*_223_, and *β*_333_. For SO_2_ molecule, index 1 corresponds to the major *O*-axis, 3 to the minor *S*-axis and 2 to a third axis that points out of the molecular plane^45,46^.

We evaluate the interaction energy (Eq. 2) in the body-fixed-frame of principal inertia axes by transforming the field (Eq. 1) from the laboratory-fixed frame. After averaging over fast optical oscillations only terms proportional to ${\mathrm{cos}}^2\left( {\omega t} \right){\mathrm{cos}}\left( {2\omega t} \right)$ contribute, and the energy as a function of the angle *θ* is given by

3 $$U\left( \theta \right) = - {\cal E}_{20}^2a{\mathrm{cos}}(2\theta ) + {\cal E}_{10}^2{\cal E}_{20}b{\mathrm{cos}}(\theta ),$$

where *a* = (*α*_33_ − *α*_22_)/8, *b* = *β*_113_cos(*ϕ*_*L*_)/8 and ${\cal E}_{i0} = {\cal E}_i(0)$ (*i* = 1,2) are the amplitudes of the laser fields. For SO_2_ molecules, *α*_22_ < *α*_33_, and *β*_113_ > 0. The cos(2*θ*) term arises from the field interaction with the linear molecular polarizability, while the cos(*θ*) term results from the hyperpolarizability interaction. Figure 2b shows *U*(*θ*) for various *ϕ*_*L*_ values. As seen, the potential is a tilted double well with the tilt controlled by the relative phase *ϕ*_*L*_. When the relative phase is *ϕ*_*L*_ = *π*/2, then *b* = 0 and the graph of the potential is the solid-green curve (Fig. 2b). In this case, the potential is a symmetric function of *θ* in the interval [0, *π*] and its two minima are equivalent. A kick by such a potential leads to the focusing of the angular distribution at *θ* = 0,*π* shortly after the kick^22^. If *ϕ*_*L*_ ≠ *π*/2, the symmetry of the potential function is broken and the minima are no longer equivalent, manifested in the asymmetric shape of the angular distribution. Near the minima (*θ* = 0, *π*), the potential can be approximated as

4 $$U\left(\theta\right)\approx\left\{\begin{array}{*{20}{l}}\hskip -2pc \left(-A+B\right)+\kappa_{-}\theta^{2} & \theta\approx0\hfill \\ \left(-A-B\right)+\kappa_{+}\left(\theta-\pi\right)^{2} & \theta\approx\pi\hfill \end{array}\right.$$

where $$A = {\cal E}_{20}^2a$$, $$B = {\cal E}_{10}^2{\cal E}_{20}b$$ and $$\kappa _ \mp = \left[ {4A \mp B} \right]/2$$.

As seen, the functional form of *U*(*θ*) is an upward opening parabola both near *θ* = 0 and *θ* = *π*. However, the depths of these parabolas (relative difference is 2*B*), as well as their stiffnesses, $\kappa _ \mp$ (0 < *κ*_−_, *κ*_+_) differ. In addition, the maximum of the potential shifts either to the left (*ϕ*_*L*_ < *π*/2), meaning that more molecules are kicked towards *θ* = *π*, or to the right (*ϕ*_*L*_ > *π*/2), in which case more molecules are kicked towards *θ* = 0. Moreover, the focusing times at *θ* = 0,*π* depend on the stiffnesses, $\kappa _ \mp$. For example, in the case of *ϕ*_*L*_ = 0 (blue-Δ curve, Fig. 2b), *κ*_−_ < *κ*_+_ and shortly after the kick, the angular distribution first focuses at *θ* = *π*, and afterwards at *θ* = 0, resulting in a pronounced left−right asymmetry of the angular distribution at the moment of each focusing event.

The traditional measure of orientation, 〈cos*θ*〉(*t*) is almost insensitive to the sharp features of the angular distribution. It does not attain its maximal value at the moment of the highest left−right asymmetry of the distribution, but rather at the moment when the difference in the areas under the distribution curve to either side of *θ* = 0 is the largest. For this reason, in discussions of the simulation and experimental results we will report also a differential measure of the orientation (defined by Eq. 5).

In general, interaction of the OTC pulse with the polarizability (the first term of the interaction potential, Eq. 2) leads to the 3D alignment. The hyperpolarizability interaction (the second term of the interaction potential, Eq. 2) breaks the symmetry along the direction of SH. The qualitative reason behind this is the following: the FW induces (via off-diagonal hyperpolarizability elements) a dipole along the SH direction, which oscillates at frequency 2*ω*, allowing SH to couple to it. For each molecule, the relative intensities of the FW and SH should be judiciously chosen for the minor axis to be oriented along the SH polarization direction. For example, in the case of iodobenze, the intensity ratio should be in favor of the SH, in contrast to the case presented here. The degree of orientation along the SH is determined by a balance between the aligning and orienting interactions with the SH field, as described by the first and the second terms in Eq. (3), respectively. Since the aligning interaction is quadratic in ${\cal E}_{20},$ while the orienting one is linear, the orientation by this mechanism (hyperpolarizability interaction) is enhanced for relatively weaker SH field amplitude.

The above discussion is applicable to asymmetric-top molecules similar to SO_2_. For orienting molecules lacking any symmetry, the remaining symmetry along the FW polarization direction and the direction of propagation must be broken, as well. This requires either the use of a nonorthogonal superposition of FW and SH^36^ or a combination of the OTC with an additional excitation that induces orientation along one of the remaining axes^41,42^. A detailed discussion of this issue and a potential combination leading to full 3D orientation of asymmetric molecule is presented in Supplementary Note 2.

### Three-dimensional simulation

Next, we proceed to a full three-dimensional simulation of the orientation process. Consider a thermal (*T* = 18 K) ensemble of $N \gg 1$ classical asymmetric-top rigid rotors with known polarizability and hyperpolarizability tensors, which are subject to a phase-locked OTC pulse. To simulate the time-dependent rotational dynamics of the molecules, we adopt an efficient singularity-free numerical technique, where quaternions are used to parametrize the rotation^47,48^. The angular velocity of each molecule is obtained by numerical integration of the Euler equations^49^. The orientation in the laboratory frame of reference is retrieved from numerical integration of the angular-velocity-dependent equation of motion for the quaternion. A detailed description of this computational approach can be found in the Methods.

In our Monte Carlo simulations, we used initially isotropic ensembles of *N* = 500,000 molecules. To account for the essentially 2D character of the detection setup (Coulomb explosion by a probe pulse circularly polarized in the *YZ* plane), and to approximate the experimental measurements, the observable quantities were calculated by integrating over a sub-ensemble of molecules lying approximately in the *YZ* plane at the time of the measurement. The selection criterion was based on the experimental arrangement, and only when angles of both minor and major axes with respect to the *YZ* plane were both less than *π*/4 the molecule was taken into account. The peak intensities of the pulses were *I*_FW_ = 1.4×10^14^ W/cm^2^, *I*_SH_ = 0.3×10^14^ W/cm^2^ and the duration (FWHM) of the pulses was 120 fs, which is much shorter than the typical periods of molecular rotation.

**Fig. 3:**
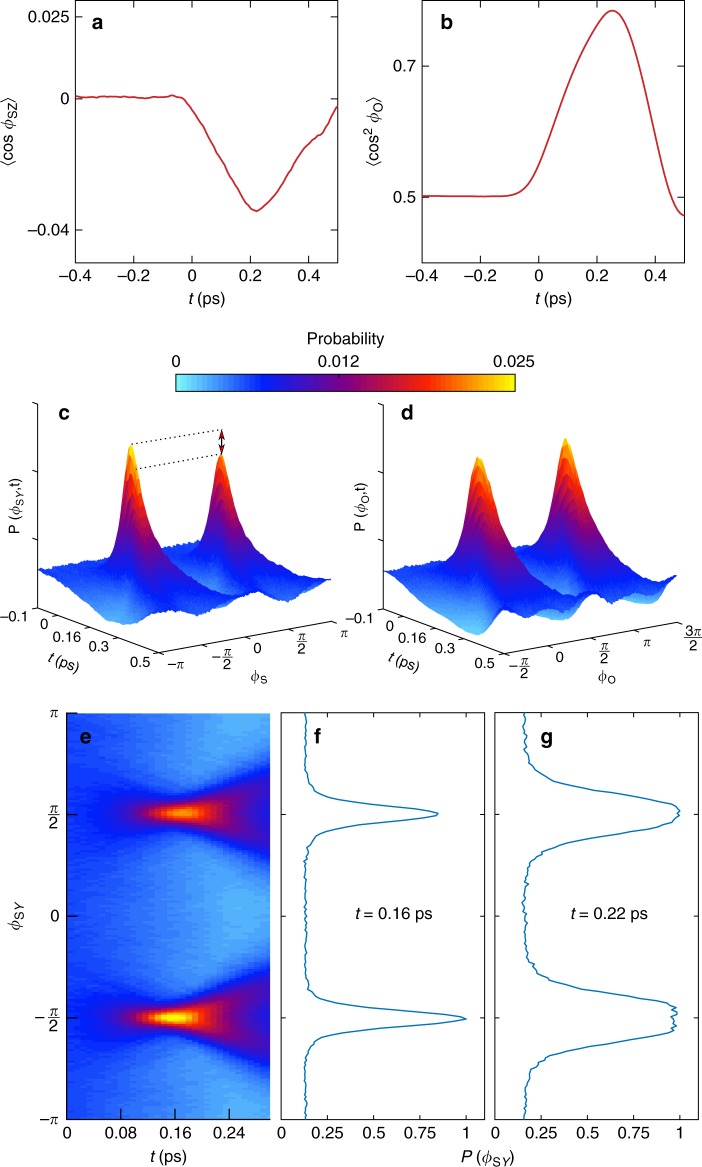
Results of three-dimensional simulation. **a** Orientation factor 〈cos*ϕ*_S*Z*_〉(*t*). **b** Alignment factor 〈cos^2^*ϕ*_O_〉(*t*). **c** 3D surface plot of the time-dependent angular distribution for *S*-axis. **d** 3D surface plot of the time-dependent angular distribution for *O*-axis. **e** Top view of the 3D surface. **f** Angular distribution *P*(*ϕ*_S*Y*_) at *t* = 0.16 ps, the moment of tighter focus around *ϕ*_S*Y*_ = −*π*/2. **g** Angular distribution of *P*(*ϕ*_S*Y*_) at *t* = 0.22 ps, the moment of maximal orientation

Fig. 3a, b shows the simulated orientation of the *S*-axis (characterized by 〈cos*ϕ*_S*Z*_〉) and alignment of the *O*-axis (characterized by 〈cos^2^*ϕ*_O_〉(*t*)) as a function of the delay with respect to the OTC pulse (*ϕ*_*L*_ = 0). Here, *ϕ*_O_ (*ϕ*_S*Z*_) is the angle that the projection of the *O*-axis (*S*-axis) on the *YZ* plane constitutes with respect to the *Y* (*Z*) laboratory axis. For an isotropic molecular ensemble, 〈cos^2^*ϕ*_O_〉(*t*) = 0.5 and 〈cos*ϕ*_S*Z*_〉 = 0.0. In the example shown in Fig. 3, both the alignment and the orientation factors reach maximal values at about 0.22 ps after the excitation by the OTC pulse. Three-dimensional surface plots of the time-dependent probability distributions *P*(*ϕ*_S*Y*_,*t*) and *P*(*ϕ*_O_,*t*) are depicted in Fig. 3c, d. Here, *ϕ*_S*Y*_ is the angle that projection of the *S*-axis on the *YZ* plane constitutes with respect to the *Y* laboratory axis. Figure 3c shows the expected asymmetric focusing of the angular distribution of the *S*-axis at *ϕ*_S*Y*_ = −*π*/2, *π*/2. In analogy to the simplified model considered above, the focusing at *ϕ*_S*Y*_ = −*π*/2 slightly precedes the one at *ϕ*_S*Y*_ = *π*/2 (see Fig. 3e). Moreover, Fig. 3f shows a pronounced asymmetry in the distribution of the *ϕ*_S*Y*_ angle at *t* = 0.16 ps (ratio of peaks’ heights is ~0.8), as reflected by the differential degree of orientation defined below in Eq. (5). The orientation factor attains its maximal value later (*t* = 0.22 ps), when the difference of the areas under the distribution curve to either side of *ϕ*_S*Y*_ = 0 is the largest (see Fig. 3g). Thus, the effect of orientation is captured by both the asymmetry of the probability distribution, *P*(*ϕ*_S*Y*_, *t*) and the integrated quantity, 〈cos*ϕ*_S*Z*_〉(*t*). Figure 3d clearly demonstrates the symmetric (*ϕ*_O_ = 0, *π*) focusing of angular distribution of the aligned *O*-axis occurring simultaneously with the orientation of the *S*-axis. The calculated maximal value of the standard orientation factor, complete ensemble-averaged projection of the *S*-axis on the direction of the SH, is ${\mathrm{cos}}(\widehat {\mathbf{S}} \cdot {\mathbf{e}}_Z) = - 0.021$.

While the optimization of the orientation process is not a set goal of the present study, it is worth mentioning that progressive improvement of the maximal degree of molecular orientation can be achieved by applying a sequence of several OTC pulses, as we discuss in Supplementary Note 1. This result is similar to earlier proposals^22,50,51^ and experimental demonstrations^52–55^ of the enhancement of molecular alignment and orientation by multiple single-color pulses. More recently, it was also shown^56^ that the splitting of a parallel two-color pulse into two sub-pulses may be beneficial for improving the overall degree of orientation of linear molecules.

### Experimental results

**Fig. 4:**
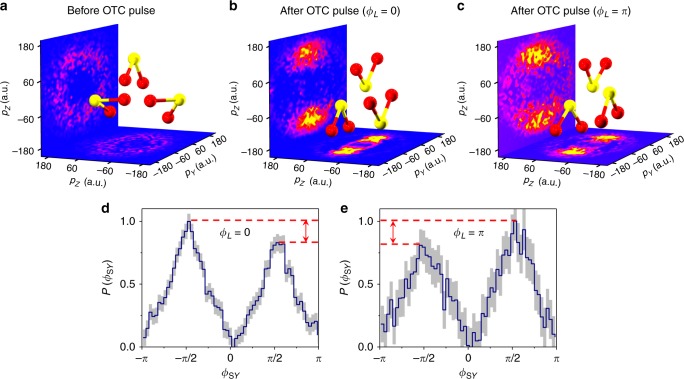
Coincidentally measured momentum distributions of S^+^ and O^+^. Here *p*_*Y*_ and *p*_*Z*_ are the projections of fragments’ momenta on *Y* and *Z* axes, respectively (measured in atomic units). **a** Isotropic momentum distribution for S^+^ and O^+^ ions measured before the arrival of OTC pulse. **b** and **c** Anisotropic momentum distributions for S^+^ and O^+^ ions measured at *t* ≈ 0.20 ps after the application of the OTC pulse at *ϕ*_*L*_ = 0 and *π*, respectively. **d** and **e** Angular distributions of *ϕ*_S*Y*_ derived from **b** and **c**, respectively. Gray bars represent the uncertainty propagated during data analysis according to Gaussian’s propagation law

We now present our experimental observations of hyperpolarizability-induced orientation by examining the data around the expected peak of the orientation at a delay of 0.20 ps. Fig. 4a–c shows the measured momentum distributions of the coincidentally measured S^+^ (vertical plane) and O^+^ (horizontal plane) ions ejected from the Coulomb-exploded triply ionized SO_2_ molecules at 0.20 ps after the application of the OTC pulse. The raw data were normalized to compensate for the detector bias and possible imperfection in the circularity of the probe pulse. The details of the data processing procedure are given in the Methods. As a reference, the momentum distribution of the ions ejected from molecules exploded before the application of the OTC pulse (i.e. at negative time delay) is presented in Fig. 4a. In this case, an isotropic angular distribution for both axes is clearly seen. After the application of the OTC pulse, the alignment of the major *O*-axis along the *Y* direction (FW polarization) can be seen on the horizontal plane for both laser relative phases *ϕ*_*L*_ = 0 and *ϕ*_*L*_ = *π* (Fig. 4b, c), as expected. The degree of alignment as extracted from these data points is estimated as 〈cos^2^*ϕ*_O_〉(*t*) = 0.79. For the minor *S*-axis, the picture is different: we see accumulation along the *Z* axis, which is the spatial direction of the SH polarization, and the distribution is clearly asymmetric. This asymmetry along the *Z* axis is the evidence for the laser-induced orientation of the molecular *S*-axis. Note that the orientation direction is determined by the phase *ϕ*_*L*_ between the FW and SH in the OTC pulse. Fig. 4d, e plots the corresponding angular distributions of the *S*-axis for the phases of *ϕ*_*L*_ = 0 and *π*, respectively. The orientation degree of the *S*-axis is estimated to be 〈cos*ϕ*_S*Z*_〉 = −0.069 for *ϕ*_*L*_ = 0 and 〈cos*ϕ*_S*Z*_〉 = 0.043 for *ϕ*_*L*_ = *π*, respectively. These values are in quantitative agreement with the numerical simulations.

As is well known, there exists another orientation mechanism due to ionization depletion^32,35,57^. If molecules oriented in a certain direction are ionized more than the others, this leaves the remaining neutral molecules preferentially oriented in the opposite direction. The intensity of the two-color field in our experiments is high enough to partially ionize the molecules, and we see clear evidence for ionization-depletion-induced-orientation in addition to the hyperpolarizability-induced effects discussed above.

**Fig. 5:**
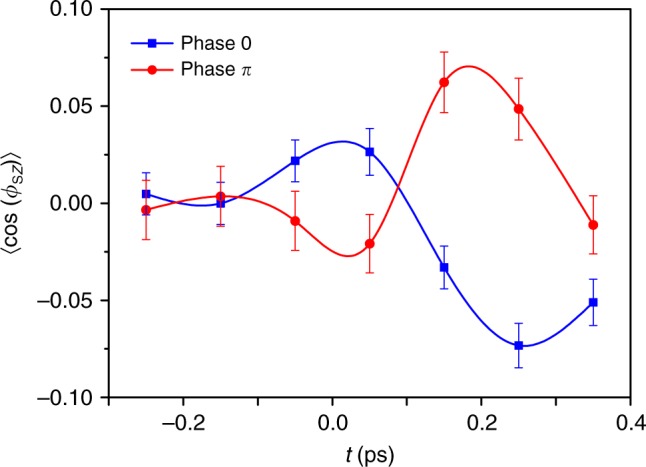
Time-dependent orientation factor, 〈cos*ϕ*_S*Z*_〉(*t*). Orientation factor as a function of a delay between OTC and the probe pulse for two relative phases *ϕ*_*L*_ = 0,*π*. Around *t* = 0, ionization leads to an increase/decrease in the orientation, while the delayed orientation peaks result from hyperpolarizability interaction. Error bars represent the uncertainty propagated during data analysis according to Gaussian’s propagation law

Figure 5 shows the measured orientation factor, 〈cos*ϕ*_S*Z*_〉(*t*) as a function of the time delay relative to the OTC pulse for the two phases, *ϕ*_*L*_ = 0 and *π*. Both curves exhibit two extrema. The maximum (minimum) around 0.2 ps stems from the hyperpolarizability interaction as discussed above, and portrayed in Figures. 3a and 5. The peak (dip) near *t* = 0, not discussed so far in this article, results from the instantaneous ionization by the OTC pulse.

**Fig. 6:**
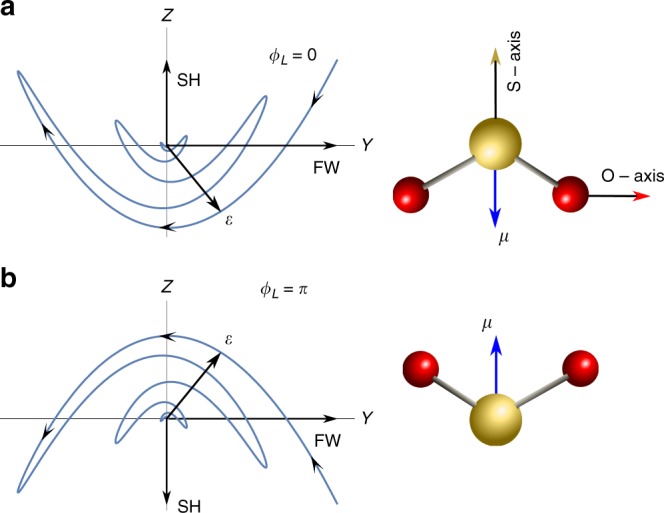
Orientation geometry for two phases. Schematic representation of trajectory of the OTC field vector, ${\cal E}$ for **a** *ϕ*_*L*_ = 0 and **b** *ϕ*_*L*_ = *π*. In both cases, SO_2_ molecule is shown in the orientation providing the maximal ionization. Blue arrows denote the molecular permanent dipole, ***μ***

In our case, the two-color electric field of the OTC pulse lacks reflection symmetry about *Y* axis in the *YZ* plane (see Fig. 6). The electronic density in SO_2_ molecule shown in Fig. 6 is mostly localized on the Sulfur side (the permanent dipole, ***μ*** points *along* the O−S−O angle bisector). Therefore, the combined asymmetric electric field of the OTC pulse preferentially ionizes molecules with *S*-axes oriented along/against the *Z* direction (depending on *ϕ*_*L*_). Such a directional ionization of molecules produces an oriented group of molecular ions. In our experiments, orientation is detected by means of Coulomb explosion, and the probability to explode a molecular ion is higher than to explode a neutral molecule. Thus, at *t* = 0, the ion signal (which we interpret as a signature of orientation) originates from the laser-produced molecular ions. After the OTC pulse, the ions’ signal is washed out because of the dispersion of their angular velocities. After proper delay, the orientation induced by the hyperpolarizability interaction dominates. The contributions of the two orientation mechanisms are well separated in time, similar to previous studies on two-color orientation of linear molecules^32,35,57^. Furthermore, in our case the signals from the two mechanisms differ in sign, providing an additional discrimination between them.

So far, the observed data have been discussed in terms of the conventional ensemble-averaged degree of orientation, 〈cos*ϕ*_S*Z*_〉(*t*). However, COLTRIMS offers additional information on the molecular angular distribution, not available in purely optical experiments (see Fig. 3f, g for the theoretical results and Fig. 4d, e for the experimental data). As seen in these figures, there is a pronounced difference in the peak values of the distribution for the left- *ϕ*_S*Y*_ = −*π*/2 and the right-facing molecules *ϕ*_S*Y*_ = *π*/2. Therefore, an additional measure of orientational asymmetry, the differential degree of orientation (DDO), may be defined as

5 $${\mathrm{DDO}} = \frac{{P(\phi _{{\mathrm{S}}Y} = \pi /2) - P(\phi _{{\mathrm{S}}Y} = - \pi /2)}}{{P(\phi _{{\mathrm{S}}Y} = - \pi /2) + P(\phi _{{\mathrm{S}}Y} = \pi /2)}}.$$

The experimentally measured results are ${\mathrm{DDO}}_{\phi _L = 0} = - 0.09$ and ${\mathrm{DDO}}_{\phi _L = \pi } = 0.11$ (Fig. 4d, e), which are close to the calculated value (≈0.10) of the maximal asymmetry of the angular distribution (Fig. 3f).

In summary, while various combinations of single and multiple ultrashort pulse excitation were shown to cause molecular alignment, in order to achieve molecular orientation, symmetry breaking must be induced. We have demonstrated, experimentally and theoretically, that all-optical field-free orientation of SO_2_ molecules, taken as a typical example of asymmetric-top molecules, can be achieved by phase-locked OTC laser pulses. The relative phase between the two fields determines the direction of the oriented molecules in space. The degree of orientation can be controlled by optimizing the parameters (peak intensity and pulse duration) of the laser fields at the FW and SH frequencies. For a given total available laser pulse energy, optimal allocation of energy to each component, and/or splitting an OTC pulse into several sub-pulses will further increase the degree of orientation, as illustrated in Supplementary Note 1. To the best of our knowledge, the present work provides the first demonstration of all-optical field-free orientation of asymmetric-top molecules in 3D space. The demonstrated scheme, possibly in combination with other available methods for molecular orientation, paves the way for angular control over asymmetric molecules, including chiral ones. Such orientational methodologies provide a new toolbox for 3D-molecular-imaging by means of X-ray free-electron laser beams with extremely high spatio-temporal resolution, and by ultra-fast electron diffraction techniques.

## Methods

### Experimental methods

Field-free 3D orientation of SO_2_ molecule in a molecular beam is induced by a pair of orthogonally polarized two-color femtosecond laser pulses. Following the orientation, an intense circularly polarized probe pulse Coulomb explodes the molecules to image their spatial orientation at various time delays, as schematically shown in Fig. 1. The output (25 fs, 790 nm, 10 kHz) of a multipass amplifier Ti:sapphire laser system is split into pump and probe arms via a beam splitter of 7:3 intensity ratio. The OTC pulse is generated in a collinear scheme by down-collimating the 70% pump beam into a 150 μm-thick *β*-barium borate (*β*-BBO) crystal to generate an SH pulse at 395 nm. To increase the doubling efficiency, a telescope is placed in front of the *β*-BBO crystal to reduce the beam diameter by a factor of two. A path of 7-mm-thick *α*-barium borate (*α*-BBO) crystals is introduced after the *β*-BBO crystal to compensate the group delay between the two colors that is induced by the optical components (wedges, mirrors and windows) along the beams’ path. The 30% probe beam is passed through a quarter wave plate, followed by a beam expander, making it circularly polarized and doubles its diameter. A motorized delay stage in the probe arm is used to synchronize and adjust its time delay with respect to the OTC pulse. The two pulses are afterwards focused onto a supersonic molecular beam of 20% mixture of SO_2_ in He in an ultrahigh vacuum chamber of the COLTRIMS apparatus by a concave silver mirror (*f* = 7.5 cm).

By pre-compensation the pulse chirp prior to the amplifier, the temporal duration of the probe pulse in the interaction region is controlled to be ~40 fs. The OTC pulse is stretched to be ~120 fs after the BBO crystals, wedge pair, the combination mirror and the entrance window. The intensities of the FW and the SH in the reaction area are measured to be *I*_FH_ ≈ 1.4×10^14^ W/cm^2^, *I*_SH_ ≈ 0.3×10^14^ W/cm^2^ and the intensity of the probe pulse is $\sim 6 \times 10^{14}\,{\mathrm{W/cm}}^{\mathrm{2}}$. The rotational temperature of the molecular beam is close to the translation temperature, which can be estimated from *T*_trans_ = Δ*p*^2^/[4 ln (4)*k*_B_*m*], where *k*_B_ is the Boltzmann’s constant, Δ*p* and *m* are the full-width at half-maximum of the momentum distribution (in the jet direction) and mass of the singly ionized ${\mathrm{SO}}_{2}^{+}$, respectively. In our experiment we measure a momentum width in the jet direction of ${\mathrm{\Delta }}p\sim 6.1\,{\mathrm{a}}{\mathrm{.u}}{\mathrm{.}}$ of ${\mathrm{SO}}_2^ +$ ions created by a laser field polarized along the *Z* axis (orthogonal to the jet direction). The rotational temperature of the SO_2_ molecule is estimated to be 18 K. The produced fragment ions are accelerated and guided by a weak homogeneous static electric field (~20 V/cm) and then detected by a time- and position-sensitive microchannel plate detector. The three-dimensional momenta of the ions are retrieved from the measured time-of-flights and positions of the impacts. Here, for the asymmetric-top molecules, the direction of the principle axes is retrieved from the coincidentally measured fragment ions of the triple-ionization-induced Coulomb explosion channel of [SO_2_ + *n*ℏ*ω* → S^+^ + O^+^ + O^+^ + 3*e*]. The angular distributions for *ϕ*_S*Y*_ and *ϕ*_O_ away from the *Y* axis at maximum 3D orientation are measured by fixing the delay stage around 0.20 ps. To increase the visibility and eliminate the bias induced by the imperfect circularity of the probe pulse, the angular distribution at negative time delay is collected as reference for the data analysis. We normalize the total probability of the angular distribution to unity for each time delay and then subtract the averaged angular distribution at negative times. Since the fragmentation of triply ionized SO_2_ molecule happens mostly in the polarization plane of the probe pulse, the data analysis is restricted to this plane by selecting molecules confined to [−*π*/4, *π*/4] with respect to the *YZ* plane.

### Numerical methods

**Fig. 7:**
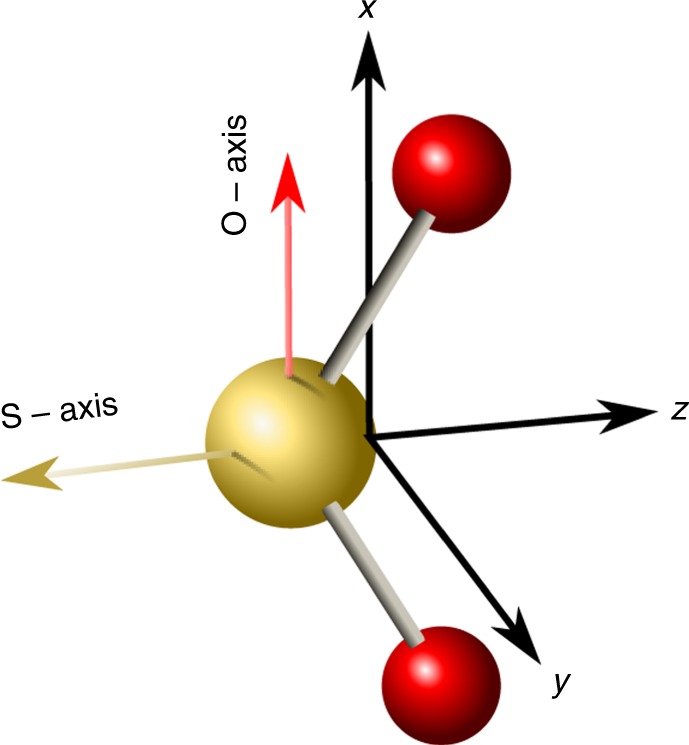
The SO_2_ molecule. Axes *x*, *y* and *z* are the principal axes of the molecule. All atoms lie in *xz* plane, oxygen is colored red, sulfur is yellow

The SO_2_ molecules are modeled as classical, rigid asymmetric tops with known anisotropic polarizability and hyperpolarizability. Figure 7 shows SO_2_ molecule with its principal axes.

In Table 1 the dynamical and electronic properties of the SO_2_ molecule are listed. Moments of inertia were computed using atomic masses and their Cartesian coordinates from ref. ^46^, *S* = (0.0,0.0,−0.682958) and *O* = (±2.333567,0.0,0.682581) (measured in Bohr).

**Table 1:** Summary of the SO_2_ properties

${\bf{I}}$ comp.	${\bf{\alpha}}$ comp.	${\bf{{\beta}}}_{ijk}$ comp.	${\bf{\mu}}$ comp.
*I*_*x*_ = 55,509	*α*_*xx*_ = 31.26	*β*_*xxz*_ = 22.0	*μ*_*x*_ = 0.0
*I*_*y*_ = 371,885	*α*_*yy*_ = 18.64	*β*_*yyz*_ = 26.5	*μ*_*y*_ = 0.0
*I*_*z*_ = 317,477	*α*_*zz*_ = 20.80	*β*_*zzz*_ = 6.4	*μ*_*z*_ = −0.79

Moments of inertia, polarizabilities, elements of the hyperpolarizability tensor and the dipole moment in the frame of principal axes (all in a.u.).

The behavior of an ensemble of $N \gg 1$ molecules is simulated using the Monte Carlo approach. We use unit quaternions to parametrize the rotation of each asymmetric top^47,48^. Quaternions are used to describe the instantaneous absolute orientation of the body-fixed frame relative to the laboratory frame and are defined by the quadruples of numbers, *q* = (*q*_0_, *q*_1_, *q*_2_, *q*_3_). The rate of change of a quaternion is given by the following differential equation

6 $$\dot q = \frac{1}{2}q{\mathrm{\Omega }},$$

where Ω = (0, **Ω**) is a pure quaternion^47,48^ derived from the angular velocity vector **Ω** = (Ω_*a*_, Ω_*b*_, Ω_*c*_) (expressed in the body-fixed frame). The two quaternions, *q* and Ω, are multiplied following the quaternions multiplication rule^47,48^. The rate of change of the angular velocity, **Ω**, is given by the Euler equations^49^,

7 $${\mathbf{I}}{\dot{{\boldsymbol{\Omega}}}}=\left({\mathbf{I}}{{\boldsymbol{\Omega}}}\right)\times{{\boldsymbol{\Omega}}}+{{\mathbf{T}}}$$

where **I** is the moment of inertia tensor and **T** = (T_*a*_, T_*b*_, T_*c*_) is the external torque. An electric field exerts torque by inducing dipole moment and interacting with it. Euler equations require the torque to be expressed in the body-fixed frame. For this, we transform the electric field, ${\cal E}$ from the laboratory frame into the body-fixed frame using the quaternion transformation rule $E = q^c{\cal E}q$, where *E* = (0, **E**), ${\cal E} = \left( {0,{\cal E}} \right)$ and *q*^*c*^ is conjugate quaternion^47,48^. There are two contributions to the torque, **T**^α ^= 〈**D** × **E**〉, where **D** = **αE** is the induced dipole moment and ${\mathbf{T}}_i^{{\beta }} = \langle {\cal E}_{ijk}{\bf{\beta }}_{jnm}{\mathbf{E}}_n{\mathbf{E}}_m{\mathbf{E}}_k\rangle ,$, where **β**_*jnm*_ is the hyperpolarizability tensor and ${\cal E}_{ijk}$ is the Levi-Civita symbol. Here, 〈⋅〉 denotes time averaging over the period of optical oscillation. Explicit expressions of torques for the case of two-color field excitation are

$${\mathbf{T}}^{\bf{\alpha }} = \frac{{{\cal E}_1^2}}{2}\left( {{\bf{\alpha }}{\mathbf{E}}_1} \right) \times {\mathbf{E}}_1 + \frac{{{\cal E}_2^2}}{2}\left( {{\bf{\alpha }}{\mathbf{E}}_2} \right) \times {\mathbf{E}}_2$$

and

$${\mathbf{T}}_i^{{\beta }} = \frac{{{\cal E}_1^2{\cal E}_2}}{4}{\cal E}_{ijk}\left[ {{\bf{\beta }}_{mnj}{\mathbf{E}}_{1m}{\mathbf{E}}_{2n}{\mathbf{E}}_{1k} + \frac{1}{2}{\bf{\beta }}_{mnj}{\mathbf{E}}_{1m}{\mathbf{E}}_{1n}{\mathbf{E}}_{2k}} \right],$$

where **E**_1_, **E**_2_ are the polarization vectors of the FW and SH, respectively, expressed in the body-fixed frame.

Quaternions describing the initial absolute orientation are generated according to the prescription given in ref. ^58^. Initial angular velocities of the thermal ensemble are distributed according to

$$f\left( {\boldsymbol{\Omega}} \right) \propto {\mathrm{exp}}\left[ { - \frac{{{\boldsymbol{\Omega}}^{T}{\mathbf{I}}{\boldsymbol{\Omega}}}}{{2k_{\mathrm B}T}}} \right] = \mathop {\prod}\limits_i {\mathrm{exp}}\left[ { - \frac{{I_i{\mathrm{\Omega }}_i^2}}{{2k_{\mathrm B}T}}} \right],$$

where *T* is the rotational temperature and *k*_B_ is the Boltzmann constant. The system of Eqs. (6) and (7) is solved numerically using the Runge−Kutta algorithm. Since this integration method does not intrinsically conserve the quaternion norm, we renormalize the quaternions at each time step^47^.

## Electronic supplementary material

Supplementary Information

## Data Availability

The data that support the findings of this study are available from the corresponding authors upon reasonable request.
