# Supplementary material for: All-optical field-free three-dimensional orientation of asymmetric-top molecules
Source: Nat Commun. 2018 Dec 3;9:5134. doi: 10.1038/s41467-018-07567-2 (PMC6277449; doi:10.1038/s41467-018-07567-2)
Supplement: Supplementary file 1 — Supplementary Information [file 41467_2018_7567_MOESM1_ESM.pdf]

All-optical Field-free Three-Dimensional Orientation of Asymmetric-Top Molecules

Lin et. al

Here, we demonstrate that a sequence of several OTC pulses may lead to a progressively increasing orientation. In addition, we discuss the prospects of future applications of the proposed OTC exctitation scheme to the problem of 3D orientation of molecules lacking any symmetry.

Supplementary Note 1: Excitation by Multiple OTC Pulses

Figure 3a shows the calculated orientation factor (for relative phase, $\phi_L = 0$) of the projection of S-axis on the Z axis, $\langle \cos \phi_{SZ} \rangle (t)$. It reaches its minimal value of about -4% at $t \approx 0.22$ ps. In [1], it was theoretically shown that the splitting of a two-color pulse into two subpulses may be beneficial for improving the overall degree of orientation. This is similar to earlier proposals [2–4] and experimental demonstrations [5–7] of the enhancement of molecular alignment and orientation by multiple single-color pulses.

Here, we show that application of a sequence of two or three OTC pulses improves the overall degree of orientation substantially (see Sup. Fig. 1).

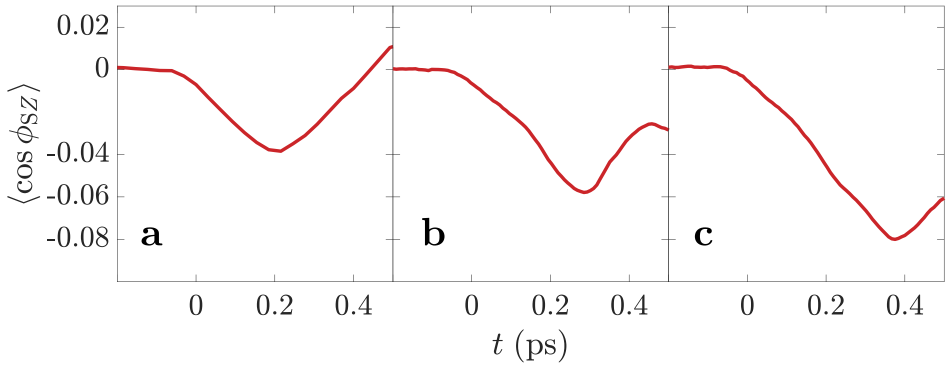

Supplementary Figure 1: **Orientation factor $\langle \cos \phi_{SZ} \rangle (t)$ for multipulse excitation** **a**, single OTC pulse, **b**, two OTC pulses, the second one is applied at $t = 0.20$ ps, **c**, three OTC pulses, the third one is applied at $t = 0.35$ ps.

The intensities of all the pulses are similar to those reported in the main text, and time delays between the pulses were chosen such that the next pulse is apllied just before the maximal orientation achieved by the previous ones. As seen from Sup. Fig. 1, several-fold increase of the maximal orientation factor is achieved, and optimization of the sequence may enhance the orientation even futher.

Supplementary Note 2: Extension to Molecules Lacking any Symmetry

The SO_2 molecule considered in our paper belongs to the C_{2v} symmetry group, which implies that alignment of the O-axis (having highest polarizability) combined with orientation of the S-axis (having second highest polarizability) is sufficient to claim 3D orientation of the molecule. In case of more complex molecules lacking any symmetry (for example, some chiral molecules), alignment of one and orientation of a another one axis is not enough to fully specify the absolute orientation of the molecules with respect to the laboratory frame. For such molecules, it is necessary to fix two principal axes, however a single OTC pulse is insufficient for this task.

The reason behind this may be understood by analyzing the minima of the interaction potential (Eq. 2 in the main text). To carry out the analysis, we parametrize the potential using three Euler angles. Here, we use the extrinsic definition of Euler angles in which the orientation of the molecular frame is reached via a sequence of three rotations about laboratory fixed axes, i.e.: rotation about laboratory Z axis by angle $\alpha \in [0, 2\pi)$, rotation about laboratory Y axis by angle $\beta \in [0, \pi]$, and finally, rotation about the laboratory Z axis by angle $\gamma \in [0, 2\pi)$. Thus, a rotation matrix that brings the molecular frame starting from the laboratory fixed frame to an arbitrary orientation is

$$\mathbf{R}\left(\alpha,\beta,\gamma\right) = \mathbf{R}_Z\left(\gamma\right)\mathbf{R}_Y\left(\beta\right)\mathbf{R}_Z\left(\alpha\right),$$

where $\mathbf{R}_Y$ and $\mathbf{R}_Z$ are the canonical active rotation matrices about laboratory Y and Z axes, respectively. Here, our choice of the molecular frame is the frame of principal axes of polarizability. In this basis, the polarizability matrix is diagonal, and the three principal axes 1, 2 and 3 are defined according to descending order of polarizabilities. Substitution of the orthogonal two-color field (with intensity ratio FW/SH > 1) and analysis of the minima of polarizability interaction $U_{\text{pol}}\left(\alpha,\beta,\gamma\right) = -\left(1/2\right)\boldsymbol{\alpha}_{ij}\mathbf{E}_i\mathbf{E}_j$ reveal the four equivalent minima

$$U_{\text{pol}}\left(0,0,0\right) = U_{\text{pol}}\left(\pi,\pi,0\right) = U_{\text{pol}}\left(\pi,0,0\right) = U_{\text{pol}}\left(0,\pi,0\right),$$

corresponding to the configurations shown in Sup. Fig. 2.

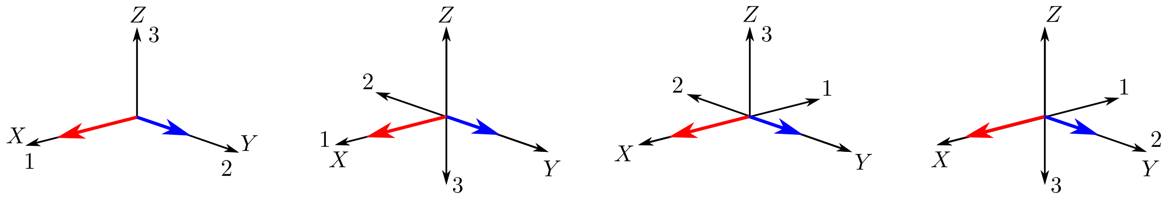

Supplementary Figure 2: **Four equivalent configurations of the frame of principal polarizability axes**. Red and blue arrows correspond to polarizations of FW and SH, respectively.

As may be seen, polarizability interaction leads to 3D molecular alignment. When the hyperpolarizability interaction is included, the four minima are no longer equivalent, and the degeneracy is partially lifted

$$U\left(0,0,0\right) = U\left(0,\pi,0\right) = -\frac{1}{8}\beta_{112}\mathcal{E}_1^2\mathcal{E}_2; \qquad U\left(\pi,0,0\right) = U\left(\pi,\pi,0\right) = \frac{1}{8}\beta_{112}\mathcal{E}_1^2\mathcal{E}_2. \tag{1}$$

Here we neglected the slight shift of the minima locations, which is justified for the intensities used in our experiment. Within each pair of configurations (first and fourth, second and third configurations of Sup. Fig. 2), the most polarizable axes (1) point in opposite directions, thus canceling the net orientation. The same is true for the least polarizable axes (3). Nevertheless, one of the pairs in 1 has the lowest potential energy, implying partial orientation of the second most polarizable axis along/against the direction of SH (depending on the sign of β_{112}).

In case of other asymmetric-top molecules similar to SO_2, the ratio FW/SH may require rearrangement. For example, in the case of iodobenzene the c_2 axis is the most polarizable one (in contrast to the SO_2 molecule in which c_2 axis is the second most polarizable). Therefore, molecular orientation requires the ratio FW/SH < 1 so that c_2 axis becomes aligned along the SH direction.

In case of molecules lacking any symmetry, 3D orientation requires breaking the remaining interaction potential degeneracy (see Sup. Eq. 1). This can be done either by changing the $\pi/2$ angle between the FW and the SH (as was done in [8]), or combing the OTC scheme with another mechanism for partial molecular orientation.

Here, we show the results of combining the OTC excitation scheme with the recently proposed method for orienting chiral molecules by laser fields with twisted polarization [9, 10].

We first apply a single-color linearly polarized pulse at $\pi/4$ angle to the X axis in the XY plane. As a result of this excitation, the most polarizable molecular axis aligns along the polarization direction of the field. At the moment of maximal alignment, we apply an OTC pulse with FW polarized along X and SH polarized along Y. In this case, the effect of the OTC pulse is three-fold. First, it interacts with the polarizability, which causes unidirectional rotation of the most polarizable axis in the XY plane. In addition, it induces a torque along the most polarizable axis according to the mechanism described in [9]. This results in the partial orientation along the propagation direction. Finally, OTC pulse interacts with the molecular hyperpolarizability resulting in a partial orientation along/against the SH direction.

We consider the application of such a sequence of pulses to a cold ensemble of propylene oxide molecules. The intensity of the first pulse is $I_{\mathrm{LP}} = 10^{14}$ W/cm^2. The OTC pulse is applied at $t = 0.31$ ps with intensities $I_{\mathrm{FW}} = 10^{14}$ W/cm^2 and $I_{\mathrm{SH}} = 0.9 \times 10^{14}$ W/cm^2. The duration of all pulses is 0.12 ps (FWHM). Sup. Figure 3 shows the resulting two orientation factors, namely the projection of the second most polarizable axis on the SH direction, and the projection of the least polarizable axis on the propagation direction.

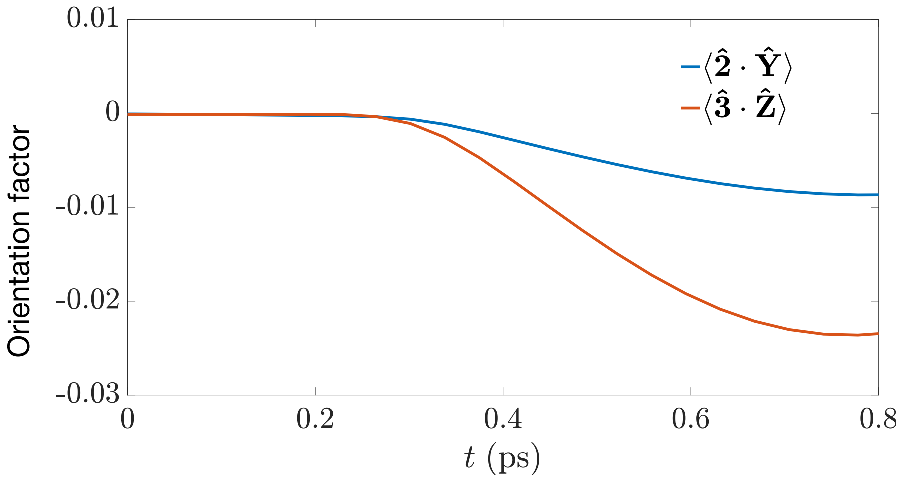

Supplementary Figure 3: **Orientation of propylene oxide molecules**. Excitation by a linearly polarized pulse combined with a delayed OTC pulse.

Supplementary Table 1 summarizes the molecular polarizability components and the moments of inertia for the propylene oxide molecule.

Moments of inertia	Polarizability tensor components
$I_a = 180386$	$\alpha_{aa} = 45.63$ $\alpha_{ab} = 2.56$
$I_b = 493185$	$\alpha_{bb} = 37.96$ $\alpha_{ac} = 0.85$
$I_c = 553513$	$\alpha_{cc} = 37.87$ $\alpha_{bc} = 0.65$

Supplementary Table 1: **Summary of molecular properties**. Eigenvalues of the moment of inertia tensor (atomic units) and components of the static polarizability tensor (atomic units) in the body-fixed frame of inertia principal axes. Computed using Gaussian software, method: CAM-B3LYP/aug-cc-pVTZ [11].

Supplementary Table 2 summarizes the molecular hyperpolarizability components.

$ij\backslash k$	a	b	c
aa	32.18	−19.73	10.79
ab		3.12	−0.057
ac			8.61
bb		−17.77	−2.38
bc			−23.66
cc			7.05

Supplementary Table 2: **Static hyperpolarizability components**. Component of $\boldsymbol{\beta}_{ijk}$ expressed in the body-fixed frame of inertia principal axes, (atomic units). Computed using Gaussian software, method: CAM-B3LYP/aug-cc-pVTZ [11].

In the frame of the principal polarizability axes, $\beta_{112} < 0$ for the propylene oxide, making the second and the third configurations shown in Sup. Fig. 2 lower in energy than the other two. This is the reason for the negative orientation factor, $\langle \mathbf{2} \cdot \mathbf{Y} \rangle$ as seen in Sup. Fig. 3.

To summarize, the OTC excitation scheme combined with an additional cross-polarized pulse holds the potential for 3D orientation of asymmetric-top molecules lacking any symmetry.

Supplementary References

[1] Zhang, S., Lu, C., Jia, T., Wang, Z. & Sun, Z. Field-free molecular orientation enhanced by two dual-color laser subpulses. *J. Chem. Phys.* **135**, 034301 (2011).

[2] Averbukh, I. S. & Arvieu, R. Angular focusing, squeezing, and rainbow formation in a strongly driven quantum rotor. *Phys. Rev. Lett.* **87**, 163601 (2001). URL `https://link.aps.org/doi/10.1103/PhysRevLett.87.163601`.

[3] Leibscher, M., Averbukh, I. S. & Rabitz, H. Molecular alignment by trains of short laser pulses. *Phys. Rev. Lett.* **90**, 213001 (2003). URL `https://link.aps.org/doi/10.1103/PhysRevLett.90.213001`.

[4] Leibscher, M., Averbukh, I. S. & Rabitz, H. Enhanced molecular alignment by short laser pulses. *Phys. Rev. A.* **69**, 013402 (2004). URL https://link.aps.org/doi/10.1103/PhysRevA.69.013402.

[5] Lee, K. F. *et al.* Two-pulse alignment of molecules. *J. Phys. B: At., Mol. Opt. Phys.* **37**, L43 (2004). URL http://stacks.iop.org/0953-4075/37/i=3/a=L02.

[6] Bisgaard, C. Z., Poulsen, M. D., Péronne, E., Viftrup, S. S. & Stapelfeldt, H. Observation of enhanced field-free molecular alignment by two laser pulses. *Phys. Rev. Lett.* **92**, 173004 (2004). URL https://link.aps.org/doi/10.1103/PhysRevLett.92.173004.

[7] Pinkham, D., Mooney, K. E. & Jones, R. R. Optimizing dynamic alignment in room temperature co. *Phys. Rev. A.* **75**, 013422 (2007). URL https://link.aps.org/doi/10.1103/PhysRevA.75.013422.

[8] Takemoto, N. & Yamanouchi, K. Fixing chiral molecules in space by intense two-color phase-locked laser fields. *Chem. Phys. Lett.* **451**, 1 – 7 (2008). URL http://www.sciencedirect.com/science/article/pii/S0009261407015655.

[9] Gershnabel, E. & Averbukh, I. S. Orienting asymmetric molecules by laser fields with twisted polarization. *Phys. Rev. Lett.* **120**, 083204 (2018). URL https://link.aps.org/doi/10.1103/PhysRevLett.120.083204.

[10] Tutunnikov, I., Gershnabel, E., Gold, S. & Averbukh, I. S. Selective orientation of chiral molecules by laser fields with twisted polarization. *J. Phys. Chem. Lett.* **9**, 1105–1111 (2018). URL https://doi.org/10.1021/acs.jpclett.7b03416. PMID: 29417812, https://doi.org/10.1021/acs.jpclett.7b03416.

[11] Frisch, M. J. *et al.* Gaussian 16, Revision A.03 (2016).
